# Supplementary material for: Collaborative training of medical artificial intelligence models with non-uniform labels
Source: Sci Rep. 2023 Apr 13;13:6046. doi: 10.1038/s41598-023-33303-y (PMC10102221; doi:10.1038/s41598-023-33303-y)
Supplement: Supplementary file 1 — Supplementary Information. [file 41598_2023_33303_MOESM1_ESM.docx]

**Supplemental Information**

## **Further Information on the Utilized Datasets**

**Tables S1** and **S2** show the demographics of the utilized datasets in this study as well as the different imaging findings available by each of them.

**Table S1**: **Statistics of the available chest radiograph datasets utilized in this study.** Automatic labeling system means the labels were generated using automatic natural-language-processing-based systems from radiology reports whereas manual means the images were manually labeled by radiologists.

|  | VinDr-CXR^18,19^ | ChestX-ray14^22^ | CheXpert^23^ | MIMIC-CXR^24,25^ | UKA-CXR^20^ |
| --- | --- | --- | --- | --- | --- |
| Total training frontal images | 15,000 | 86,524 | 128,356 | 210,652 | 122,294 |
| Total test frontal images | 3,000 | 25,596 | 29,320 | 2,844 | 39,824 |
| Total frontal images | 18,000 | 112,120 | 157,676 | 215,187 | 193,361 |
| Number of labels available | 28 | 15 | 14 | 14 | 9 |
| Labeling system for training set | manual | automatic | automatic | automatic | manual |
| Labeling system for test set | manual | automatic | automatic | automatic | manual |

**Table S2**: **Details of available labels corresponding to each chest radiograph dataset utilized in this study.** “No finding” indicates the absence of all other diseases.

| **Label name** | VinDr-CXR^18,19^ | ChestX-ray14^22^ | CheXpert^23^ | MIMIC-CXR^24,25^ | UKA-CXR^20^ |
| --- | --- | --- | --- | --- | --- |
| *Aortic enlargement* | yes | No | no | no | no |
| *Atelectasis* | yes | yes | yes | yes | yes |
| *Calcification* | yes | no | no | no | no |
| *Cardiomegaly* | yes | yes | yes | yes | yes |
| *Clavicle fracture* | yes | no | yes | yes | no |
| *Consolidation* | yes | yes | yes | yes | no |
| *Edema* | yes | yes | yes | yes | no |
| *Emphysema* | yes | yes | no | no | no |
| Enlarged cardiomediastinum | no | no | yes | yes | no |
| *Enlarged PA* | yes | no | no | no | no |
| *Hernia* | no | yes | no | no | no |
| *Interstitial lung disease* | yes | no | no | no | no |
| *Infiltration* | yes | yes | no | no | yes |
| *Lung cavity* | yes | no | no | no | no |
| *Lung lesion* | yes | no | yes | yes | no |
| *Lung opacity* | yes | no | yes | yes | no |
| *Lung tumor* | yes | no | no | no | no |
| *Mediastinal shift* | yes | no | no | no | no |
| *No finding* | yes | yes | yes | yes | no |
| *Nodule/Mass* | yes | yes | no | no | no |
| *Pleural effusion* | yes | yes | yes | yes | yes |
| *Pleural thickening* | yes | yes | yes | yes | no |
| *Pneumonia* | yes | yes | yes | yes | yes |
| *Pneumothorax* | yes | yes | yes | yes | yes |
| *Pulmonary fibrosis* | yes | yes | no | no | no |
| *Rib fracture* | yes | no | yes | yes | no |
| *Tuberculosis* | yes | no | no | no | no |

## **Detailed Evaluation Results of All Experiments**

Detailed evaluation results of all experiments in our study for all the individual diseases including further evaluation metrics are provided in **Tables S3–S5**.

**Table S3**: **Details of the results of the comparison between local and FFL-based training of VinDr-CXR dataset with non-overlapping labels for different training set sizes, tested on the VinDr-CXR test set.** The results show the individual AUROC values for each label, and average accuracy, sensitivity, and specificity values over all labels. The FFL was performed in combination with UKA-CXR dataset of n=122,294 images with 2 other labels including *pleural effusion right* and *pneumonic infiltrates left*.

|  | Local 2K | Local 5K | Local 15K | FFL 2K | FFL 5K | FFL 15K |
| --- | --- | --- | --- | --- | --- | --- |
| *Cardiomegaly* | 0.90 ± 0.01 | 0.89 ± 0.01 | 0.92 ± 0.01 | 0.88 ± 0.01 | 0.90 ± 0.01 | 0.94 ± 0.01 |
| *Pleural effusion* | 0.82 ± 0.02 | 0.90 ± 0.02 | 0.96 ± 0.01 | 0.91 ± 0.02 | 0.94 ± 0.01 | 0.96 ± 0.01 |
| Average accuracy | 0.78 ± 0.08 | 0.85 ± 0.03 | 0.89 ± 0.04 | 0.84 ± 0.07 | 0.88 ± 0.05 | 0.90 ± 0.03 |
| Average sensitivity | 0.80 ± 0.10 | 0.80 ± 0.04 | 0.87 ± 0.06 | 0.80 ± 0.06 | 0.81 ± 0.05 | 0.89 ± 0.03 |
| Average specificity | 0.77 ± 0.09 | 0.86 ± 0.04 | 0.89 ± 0.04 | 0.84 ± 0.08 | 0.88 ± 0.05 | 0.90 ± 0.03 |

**Table S4**: **Results of the comparison between local and FFL-based training of VinDr-CXR dataset with overlapping labels for different training set sizes, tested on the VinDr-CXR test set.** Results show the individual AUROC values for each label, and average accuracy, sensitivity, and specificity values over all labels. The FFL was performed in combination with UKA-CXR dataset of n=122,294 images with 7 other labels including *cardiomegaly*, *pleural effusion right*, *pleural effusion left*, *pneumonic infiltrates right*, *pneumonic infiltrates left*, *atelectasis right*, and *atelectasis left*.

|  | Local 2K | Local 5K | Local 15K | FFL 2K | FFL 5K | FFL 15K |
| --- | --- | --- | --- | --- | --- | --- |
| *No finding* | 0.81 ± 0.01 | 0.79 ± 0.01 | 0.83 ± 0.01 | 0.80 ± 0.01 | 0.83 ± 0.01 | 0.83 ± 0.01 |
| *Aortic enlargement* | 0.82 ± 0.01 | 0.82 ± 0.01 | 0.83 ± 0.01 | 0.81 ± 0.01 | 0.83 ± 0.01 | 0.85 ± 0.01 |
| *Pleural thickening* | 0.71 ± 0.02 | 0.75 ± 0.02 | 0.81 ± 0.02 | 0.77 ± 0.02 | 0.78 ± 0.02 | 0.77 ± 0.02 |
| *Cardiomegaly* | 0.86 ± 0.01 | 0.86 ± 0.01 | 0.90 ± 0.01 | 0.86 ± 0.01 | 0.89 ± 0.01 | 0.90 ± 0.01 |
| *Pleural effusion* | 0.84 ± 0.02 | 0.89 ± 0.02 | 0.95 ± 0.01 | 0.80 ± 0.02 | 0.85 ± 0.02 | 0.90 ± 0.02 |
| *Pneumothorax* | 0.74 ± 0.06 | 0.70 ± 0.07 | 0.85 ± 0.06 | 0.71 ± 0.07 | 0.79 ± 0.06 | 0.86 ± 0.04 |
| *Atelectasis* | 0.63 ± 0.03 | 0.71 ± 0.03 | 0.65 ± 0.03 | 0.70 ± 0.03 | 0.77 ± 0.03 | 0.79 ± 0.03 |
| Average accuracy | 0.74 ± 0.08 | 0.74 ± 0.08 | 0.76 ± 0.12 | 0.74 ± 0.07 | 0.77 ± 0.06 | 0.77 ± 0.08 |
| Average sensitivity | 0.71 ± 0.12 | 0.71 ± 0.11 | 0.78 ± 0.09 | 0.71 ± 0.15 | 0.75 ± 0.09 | 0.77 ± 0.09 |
| Average specificity | 0.74 ± 0.08 | 0.74 ± 0.09 | 0.75 ± 0.13 | 0.74 ± 0.08 | 0.76 ± 0.07 | 0.77 ± 0.09 |

**Table S5**: **Details of the results of the comparison between local and FFL-based training for 5 different datasets.** The results show the individual AUROC values for each label, and average accuracy, sensitivity, and specificity values over all labels, tested on the test benchmarks of the corresponding datasets. The FFL process for each dataset was performed in combination with the other 4 datasets including 5 different labels for each dataset. VinDr-CXR, ChestX-ray14, CheXpert, MIMIC-CXR, and UKA-CXR cohorts contained n=15,000, n=86,524, n=128,356, n=210,652, and n=122,294 training images, respectively.

| Dataset name | | Individual AUROC (in order) | Average accuracy | Average sensitivity | Average specificity |
| --- | --- | --- | --- | --- | --- |
| **VinDr-CXR** *(no finding, aortic enlargement, pleural thickening, cardiomegaly, pleural effusion)* | Local | 0.841 ± 0.008, 0.859 ± 0.011, 0.810 ± 0.017, 0.893 ± 0.011, 0.933 ± 0.013 | 0.777 ± 0.065 | 0.822 ± 0.049 | 0.763 ± 0.074 |
|  | FFL | 0.849 ± 0.008, 0.869 ± 0.011, 0.832 ± 0.016, 0.909 ± 0.009, 0.966 ± 0.007 | 0.816 ± 0.087 | 0.823 ± 0.050 | 0.801 ± 0.102 |
| **ChestX-ray14** *(cardiomegaly, effusion, pneumonia, consolidation, no finding)* | Local | 0.865 ± 0.005, 0.791 ± 0.003, 0.653 ± 0.011, 0.709 ± 0.006, 0.699 ± 0.003 | 0.655 ± 0.095 | 0.718 ± 0.093 | 0.661 ± 0.106 |
|  | FFL | 0.874 ± 0.05, 0.793 ± 0.003, 0.645 ± 0.011, 0.705 ± 0.006, 0.705 ± 0.003 | 0.658 ± 0.079 | 0.710 ± 0.105 | 0.667 ± 0.095 |
| **CheXpert** *(cardiomegaly, lung opacity, lung lesion, pneumonia, edema)* | Local | 0.877 ± 0.003, 0.723 ± 0.003, 0.771 ± 0.007, 0.743 ± 0.009, 0.866 ± 0.002 | 0.722 ± 0.059 | 0.759 ± 0.064 | 0.697 ± 0.087 |
|  | FFL | 0.876 ± 0.003, 0.726 ± 0.003, 0.769 ± 0.007, 0.751 ± 0.009, 0.863 ± 0.002 | 0.718 ± 0.063 | 0.762 ± 0.062 | 0.695 ± 0.086 |
| **MIMIC-CXR** *(enlarged cardiomediastinum, consolidation, pleural effusion, pneumothorax, atelectasis)* | Local | 0.701 ± 0.025, 0.704 ± 0.019, 0.876 ± 0.007, 0.831 ± 0.020, 0.749 ± 0.010 | 0.704 ± 0.100 | 0.723 ± 0.081 | 0.702 ± 0.110 |
|  | FFL | 0.718 ± 0.026, 0.734 ± 0.018, 0.876 ± 0.007, 0.848 ± 0.021, 0.754 ± 0.010 | 0.716 ± 0.095 | 0.744 ± 0.106 | 0.710 ± 0.106 |
| **UKA-CXR** *(pleural effusion left, pleural effusion right, cardiomegaly, pneumonic infiltrates left, pneumonic infiltrates right)* | Local | 0.920 ± 0.003, 0.940 ± 0.002, 0.855 ± 0.002, 0.935 ± 0.002, 0.930 ± 0.002 | 0.824 ± 0.031 | 0.860 ± 0.046 | 0.818 ± 0.036 |
|  | FFL | 0.925 ± 0.003**,** 0.942 ± 0.002, 0.857 ± 0.002, 0.937 ± 0.002, 0.932 ± 0.002 | 0.832 ± 0.035 | 0.858 ± 0.038 | 0.825 ± 0.044 |

## **Exemplary Radiographs from the Utilized Datasets**

**Fig. S1** illustrative radiographs from the internal UKA-CXR^20^ dataset, utilized in this study. All other data, including the images and cohort statistics (VinDr-CXR^18,19^, ChestX-ray14^22^, CheXpert^23^, and MIMIC-CXR^24,25^) are publicly available.

**
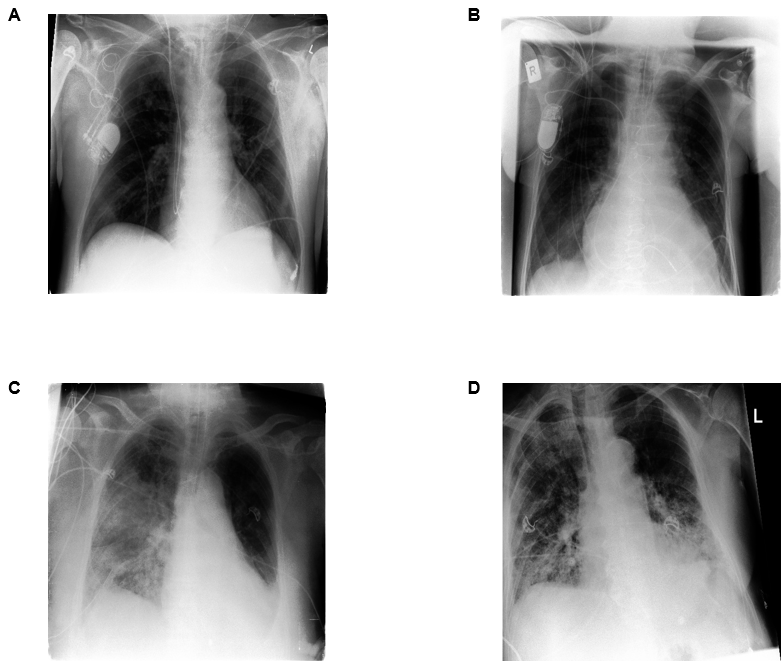
**

**Fig. S1**: **Exemplary radiographs utilized in this study.** **(A)** 70-year-old male healthy subject (radiologists reported “no finding”, i.e., no pathological changes. Cardiac pacemaker can be seen on the right patient side). **(B)** 86-year-old male patient diagnosed with *cardiomegaly*. **(C)** 71-year-old male patient diagnosed with *cardiomegaly*, *atelectasis right*, and *pneumonic infiltrates right.* **(D)** 86-year-old male patient diagnosed with *atelectasis right*, *atelectasis left, pneumonic infiltrates right,* and *pneumonic infiltrates left.*

## **Exemplary Convergence and ROC Curves**

**Fig. S2** and **Fig. S3** show exemplary training loss and receiver-operator-characteristic (ROC) curves. FFL-based training is performed with partially overlapping labels on UKA-CXR^20^ (n=122,294, labels: *cardiomegaly*, *pleural effusion right*, *pleural effusion left*, *pneumonic infiltrates right*, *pneumonic infiltrates left*, *atelectasis right*, and *atelectasis left*) and on VinDr-CXR^18,19^ (on 2K images, labels: over *no finding*, *aortic enlargement*, *pleural thickening*, *cardiomegaly*, *pleural effusion*, *pneumothorax*, and *atelectasis*). Performance tested on an independent VinDr-CXR test set.

**
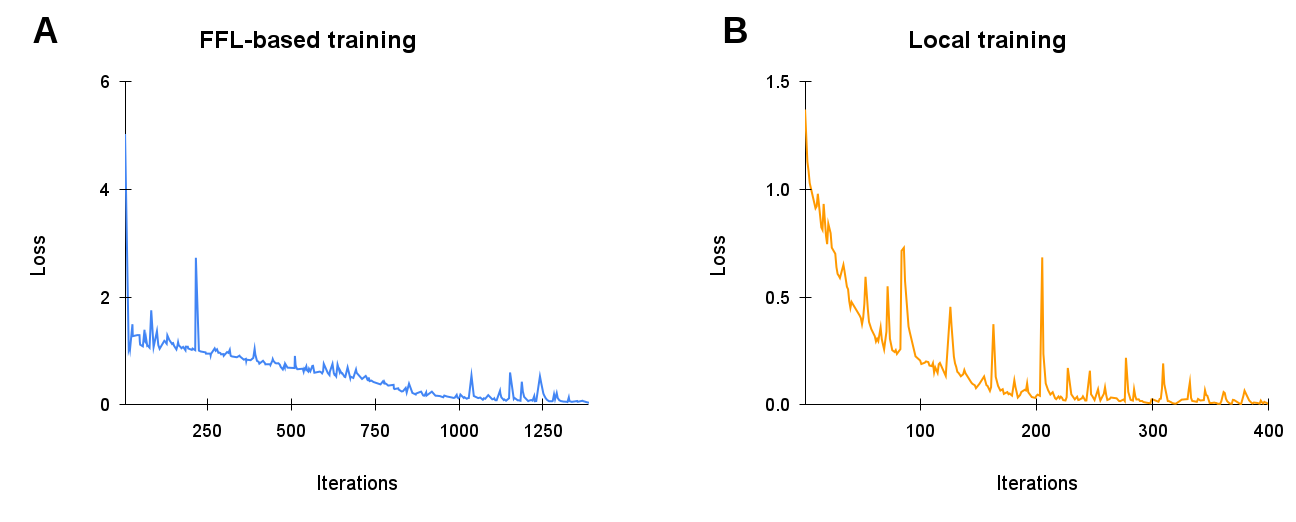
**

**Fig. S2**: **Training loss curves for FFL-based training and local training of classification models.** FFL-based training is performed with partially overlapping labels on UKA-CXR^20^ (n=122,294, labels: *cardiomegaly*, *pleural effusion right*, *pleural effusion left*, *pneumonic infiltrates right*, *pneumonic infiltrates left*, *atelectasis right*, and *atelectasis left*) and on VinDr-CXR^18,19^ (on 2K images, labels: over *no finding*, *aortic enlargement*, *pleural thickening*, *cardiomegaly*, *pleural effusion*, *pneumothorax*, and *atelectasis*). **(A)** FFL-based training, **(B)** *Local training.*

**
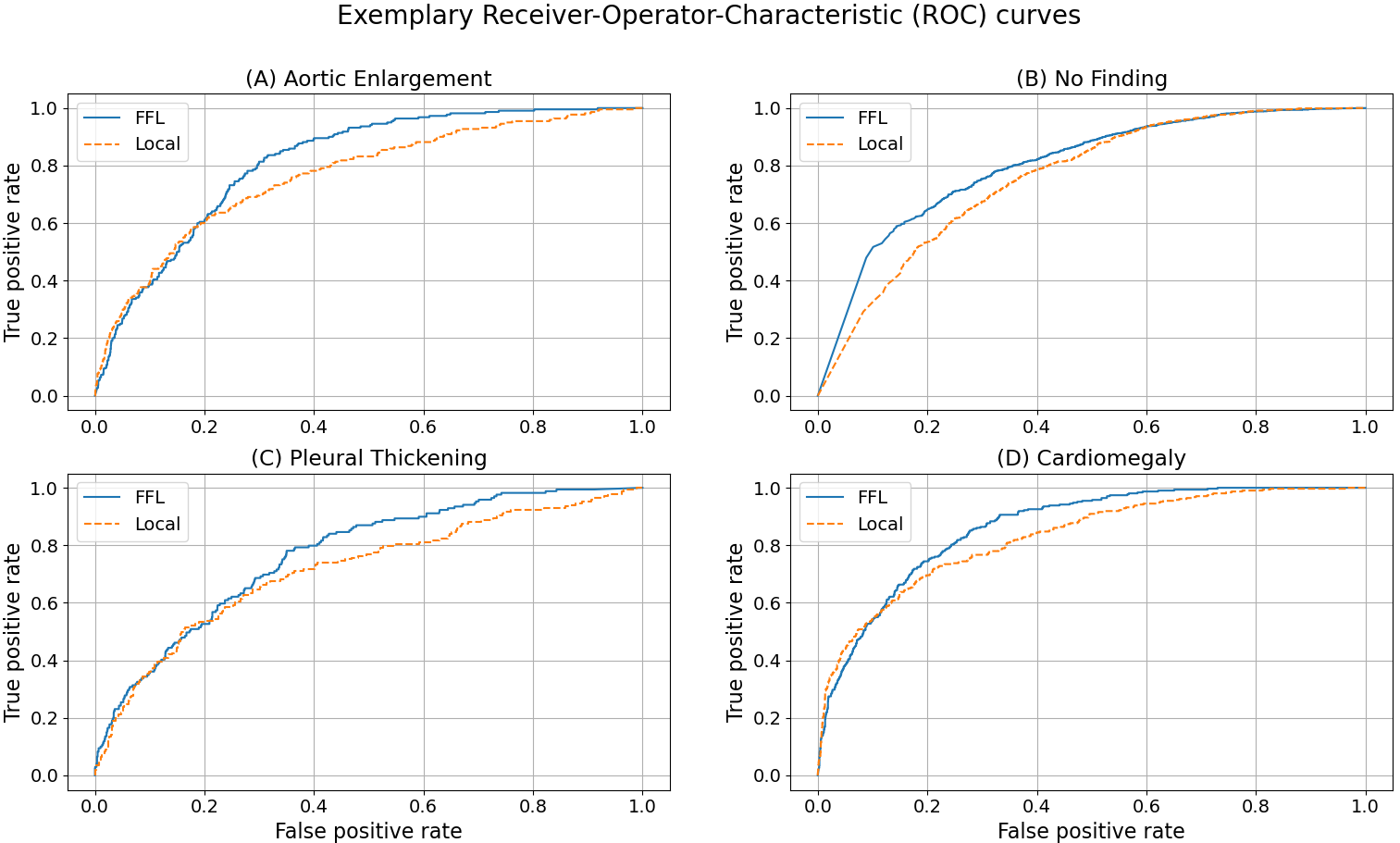
**

**Fig. S3**: **Comparison between FFL-based training and local training of classification models in terms of receiver-operator-characteristic (ROC) curves.** FFL-based training is performed with partially overlapping labels on UKA-CXR^20^ (n=122,294, labels: *cardiomegaly*, *pleural effusion right*, *pleural effusion left*, *pneumonic infiltrates right*, *pneumonic infiltrates left*, *atelectasis right*, and *atelectasis left*) and on VinDr-CXR^18,19^ (on 2K images, labels: over *no finding*, *aortic enlargement*, *pleural thickening*, *cardiomegaly*, *pleural effusion*, *pneumothorax*, and *atelectasis*). Performance tested on an independent VinDr-CXR test set. ROC curves are illustrated for individual labels including **(A)** aortic enlargement, **(B)** *no finding*, **(C)** pleural thickening, and **(D)** *cardiomegaly*.
